# Flow-diverting devices in the treatment of unruptured ophthalmic segment aneurysms at a mean clinical follow-up of 5 years

**DOI:** 10.1038/s41598-021-87498-z

**Published:** 2021-04-28

**Authors:** Przemysław Kunert, Katarzyna Wójtowicz, Jarosław Żyłkowski, Maciej Jaworski, Daniel Rabczenko, Jakub Wojciechowski, Kamil Leśniewski, Andrzej Marchel

**Affiliations:** 1 Department of Neurosurgery, Medical University of Warsaw, Banacha str. 1a, 02-097 Warszawa, Poland; 2 Second Department of Radiology, Medical University of Warsaw, Warsaw, Poland; 3 Department of Population Health Monitoring and Analysis, National Institute of Public Health - National Institute of Hygiene, Warsaw, Poland

**Keywords:** Cerebrovascular disorders, Outcomes research

## Abstract

A shift toward the endovascular treatment of ophthalmic segment aneurysms is noticeable. However, it is not clear if the long-term treatment results improve with the development of endovascular methods. The aim of this study was to present the outcomes of the treatment of unruptured ophthalmic aneurysms using flow diverting devices (FDD) with or without coiling. This retrospective study included 52 patients with 65 UIAs treated in 2009–2016. The mean aneurysm size was 8.8 mm. Eight aneurysms were symptomatic. Therapeutic procedures included: 5 failed attempts, 55 first sessions with FDD deployment (bilateral procedures in 3) and 3 retreatment procedures. To cover 55 ICAs, 25 Silk, 26 Pipeline, 9 Fred and 1 Surpass FDD were used. FDD with coiling was applied in 19(29.2%), mainly for symptomatic and larger aneurysms. Mean radiological and clinical follow-up was 12 and 61 months, respectively. Postprocedural deterioration was noted in 3(5.8%) patients, but in long-term the modified Rankin Scale grades 0–2 were achieved in 98.1% of patients. One patient died from the treated aneurysm rupture (annual risk—0.07%). Raymond–Roy occlusion classification class I or II was achieved in 98.5% in the long term, with similar results in both groups. Complications occurred in 40.4% of patients and the most frequent were: imperfect FDD deployment (15%), failed attempt of FDD deployment (9.6%) and late FDD stenosis (9.6%). Flow-diverting devices, with additional coiling in selected cases, may offer a very high proportion of satisfactory outcomes. However, in our experience the high risk of complications remains.

## Introduction

The ophthalmic segment of internal carotid artery (ICA-C6), starts at the distal dural ring and ends just before the posterior communicating artery origin^1^. Aneurysms of ICA-C6 occur with a frequency of approximately 5% of all intracranial aneurysms^2^. Besides the subarachnoid haemorrhage, the clinical presentations include visual deficits^3^. Surgical clipping and different endovascular techniques like coil embolisation, flow diverting device (FDD) deployment and combination of coiling with stenting are current options for the treatment of ICA-C6 aneurysms^4–6^. Permanent morbidity ranges from 0.8 to 38%^4,6–11^, and the new visual deficits occur more often after clipping than endovascular treatment^5,11–14^. Flow diverting stents are promising devices for a higher percentage of fully occluded aneurysms as compared to coil embolisation^15^.

The aim of this study was to present the clinical and radiological outcomes of endovascular treatment of unruptured ICA-C6 aneurysms using FDDs with or without coiling. Different factors were tested as potential predictors of the most favourable outcome.

## Material and methods

### Patients

This retrospective study included 52 patients with 65 unruptured aneurysms (UIA) located on ICA-C6 treated in 2009–2016. The mean patient age was 52.5 years (range: 26–76 years old). Forty-five (86.5%) patients were female. Four (7.7%) patients had a history of subarachnoid haemorrhage (SAH) from another aneurysm. Twenty-seven (51.9%) patients were smokers, 26 (50%) patients had arterial hypertension and 7 (13.5%) had a positive family history of intracranial aneurysms.

All study participants underwent regular, neurosurgical management and treatment. Afterwards, anonymized patient data was retrospectively analyzed by physicians who have access to patients’ medical records. The Bioethics Committee of the Medical University of Warsaw (local institutional review board) approved this study and waived the need for informed consent. Moreover, the study has been performed in accordance with the ethical standards as laid down in the 1964 Declaration of Helsinki.

The mean radiological follow-up was 12 months (range: 1 to 24 months), and the mean clinical follow-up was 61 months (range: 3 to 111 months). The follow-up data were available for all patients.

### Aneurysms

Of 65 aneurysms, 39 (60%) were ophthalmic, 24 (36.9%) superior hypophyseal artery and 2 (3.1%) were dorsal ICA (blister-like) aneurysms. Eleven (21.2%) patients had more than one treated ICA-C6 aneurysm: unilaterally in 8 and bilaterally in 3 cases. The measurements of dome and neck in 2 blister-like aneurysms were feasible therefore they were analysed together with saccular aneurysms. According to the convention^16^, 26 (40%) aneurysm necks were small (≤ 4 mm) and 39 (60%) were large (> 4 mm); dome-to-neck ratio was ≤ 2 in 44 (67.7%) aneurysms and > 2 in 21 (32.3%) aneurysms (Table 1).

**Table 1:** Aneurysm characteristics in whole group and subgroups, according to the treatment technique.

	All aneurysms	Aneurysms treated with FDD (N^o^46)	Aneurysms treated with FDD + C (N^o^19)	*p*
**Aneurysm**				
Mean size (SD)	8.8 mm (5.4)	6.8 mm (3.0)	13.5 mm (7.0)	0.001
Size range	2.2–28 mm	2.2–18 mm	3.3–28 mm	
Aneurysm size ≤ 7 mm	32 (49.2%)	28 (60.9%)	4 (21.1%)	< 0.001
Aneurysm size 7–12 mm	22 (33.8%)	17 (37.0%)	5 (26.3%)	
Aneurysm size 13–24	9 (13.8%)	1 (2.2%)	8 (42.1%)	
Aneurysm size ≥ 25 mm	2 (3.1%)	0	2 (10.5%)	
**Neck**				
Mean size (SD)	4.7 mm (1.9)	4.3 mm (1.6)	5.9 mm (2.0)	0.004
Size range	1.4–10 mm	1.4–8.8 mm	2.9–10 mm	
**Dome-to-neck ratio**				
Mean (SD)	1.8 (0.7)	1.7 (0.7)	2.2 (0.7)	0.009
Range	0.7–3.8	0.7–4.1	1.0–3.5	
Symptomatic	8 (12.3%)	3 (6.5%)	5 (26.3%)	0.082

FDD, flow diverting device alone; FDD+C, flow diverting device with coiling; SD,–standard deviation.

Symptoms. Eight (12.3%) aneurysms were symptomatic, with multiple symptoms in 4. Visual field deficit was present in 6 patients, acuity decrease in 4, optic nerve atrophy in 1 and oculomotor nerve palsy in 1. The causes of neuroimaging in the remaining patients were headache (N^o^11; 21.2%), dizziness (N^o^7; 13.5%), SAH from another aneurysm (N^o^4; 7.7%) or non-specific complaints (N^o^22; 42.3%).

### Procedures

52 patients underwent 63 therapeutic procedures for 65 UIAs. Therapeutic procedures included: 5 failed attempts, 55 first sessions with FDD deployment (including 3 patients with bilateral procedures) and 3 retreatment procedures. In total, there were 182 invasive procedures, including 119 DSAs.

Twenty-five Silk, 26 Pipeline, 9 Fred and 1 Surpass FDD were used to cover 55 ICAs in 52 patients. FDD with additional coiling (FDD + C) was performed in 19 (29.2%) cases.

### Methods

Data on demographics, medical history, symptoms, aneurysms, treatment, complications and results were recorded from patient files. The follow up data were collected based on files from control hospital stays, ambulatory visits and telephone conversations. Indications for and its technique treatment were discussed by the whole team including neurosurgeons and interventional radiologists. All procedures were performed by 2 interventional radiologists (MJ, JŻ). The clinical and radiological outcomes were assessed by neurosurgeons (KW, PK, JW, KL, AM).

Follow-up Dyna-CT was performed 3 months after the procedure in the last 28 patients of the series. Routinely, the patients underwent the follow-up DSA 6 and 12 months after the procedure. Further imaging was scheduled individually only for patients with incompletely occluded aneurysms. The Raymond–Roy (RROC) and O'Kelly-Marotta (OKM) grading scales were used for aneurysm occlusion assessment^17,18^. The clinical status was established with the use of the modified Rankin Scale (mRS). Formal ophthalmologic evaluation was available for all patients with symptomatic UIAs. The clinical outcomes were calculated per patient, radiological outcomes per aneurysm, and complication risk per patient, per aneurysm and per procedure.

### Statistical methods

Assessment of the differences between groups was done using t-test for numerical and chi-square or Fisher exact test for categorical data. Potential determinants of the outcome were assessed using one and multivariate logistical regression models, using R 3.5.1 statistical software (R: A Language and Environment for Statistical Computing, R Core Team, Vienna, Austria, https://www.R-project.org).

### Ethics approval

All study participants underwent regular, neurosurgical management and treatment. Afterwards, anonymized patient data was retrospectively analyzed by physicians who have access to their patients’ medical records. For that reason, a signed consent from each patient, or legal representative, was not deemed necessary (Declaration of Helsinki, 25). The Bioethics Committee of the Medical University of Warsaw (local institutional review board) approved this study and waived the need for informed consent.

## Results

### Clinical outcomes

On admission, 8 (15.5%) patients presented with vision symptoms (mRS grade 1) and 1 (1.9%) with cognitive impairment (mRS grade 2) after SAH in the past (Table 2).

**Table 2:** Clinical and radiological outcomes.

mRS	On admission	On postprocedural day 1	At discharge	In the most recent follow-up	
**Clinical outcomes according to the modified Rankin Scale (mRS)**					
Grade 0	43 (82.7%)	41 (78.8%)	42 (80.8%)	41 (78.8%)	Satisfactory outcome-51 (98.1%)
Grade 1	8 (15.5%)	8 (15.4%)	8 (15.4%)	9 (17.3%)	
Grade 2	1 (1.9%)	1 (1.9%)	1 (1.9%)	1 (1.9%)	
Grade 3	0	1 (1.9%)	0 (%)	0	Unsatisfactory outcome-1 (1.9%)
Grade 4	0	1 (1.9%)	1 (1.9%)	0	
Grade 5	0	0	0	0	
Grade 6	0	0	0	1 (1.9%)	

	RROC Class I	RROC Class II	RROC Class III		
**Radiological outcomes according to the Raymond–Roy occlusion classification**					
Immediate post-procedural result					
FDD alone	3 (6.5%)	4 (8.7%)	39 (84.8%)	*p* = 0.04	
FDD + C	5 (26.3%)	2 (10.5%)	12 (63.2%)		
All aneurysms	8 (12.3%)	6 (9.2%)	51 (78.5%)		
Results at 6 months follow-up DSA					
FDD alone	35 (76.1%)	4 (8.7%)	7 (15.2%)	p-NS	
FDD + C	12 (66.7%)	2 (11.1%)	4 (22.2%)		
All aneurysms	47 (73.4%)	6 (9.4%)	11 (17.2%)		
Long-term results (the last follow-up DSA)*					
FDD alone	44 (95.7%)	2 (4.3%)	0	p-NS	
FDD + C	18 (94.7%)	0	1 (5.3%)**		
All aneurysms	62 (95.4%)	2 (3.1%)	1 (1.5)**		

NS, not significant.

*Include also results of retreatment.

**In patient who died, the last DSA performed after SAH revealed a critical distal end stent stenosis and aneurysm remnant (RROC class III, OKM B).

On the postprocedural day one, deterioration was noted in 3 (5.8%) patients including minor deficit in 2 and major in 1. One patient had prolonged cognitive impairment due to thromboembolic complications (mRS grade 4). At the discharge from hospital 51 (98.1%) patients were stable with their mRS scores as before the treatment.

In follow-up, 49 patients were stable and 1 patient with postprocedural cognitive impairment significantly improved. One patient died from the treated aneurysm rupture. One patient deteriorated for a couple of months to mRS grade 3 because of thromboembolic complications at the 6-month follow-up DSA and she eventually improved to mRS grade 1. In the most recent follow-up, 98.1% of patients achieved satisfactory clinical outcome (mRS grades 0–2).

The overall post-treatment risk of aneurysm rupture was 0.33% (1/304 aneurysm-years) during a mean follow-up of 61 months. The annual risk of aneurysm rupture after treatment reached 0.07%. The overall permanent morbidity (mRS > 2) and mortality rate was 1.9%. However, if all minor (also visual) and transient post-procedure deficits were included, then the combined morbidity and mortality was 17.3% (9/52).

Visual symptoms were stable in follow-up in 4 (50%) patients. One (12.5%) patient had transient and two (25%) permanent deterioration after treatment. The vision improved only in one (12.5%) patient. Patients with asymptomatic UIAs had no visual complications immediately after treatment.

### Radiological outcomes

Immediate result of the first procedure. The complete occlusion was demonstrated in only 8 (12.3%) aneurysms at the end of the procedure (Table 2). However, the immediate postprocedural results were significantly better when the additional coiling was applied (RROC class I + II; 36.8% for FDD + C vs. 15.2% for FDD; *p* = 0.04).

The patient, who died 3 months post-procedure due to SAH from the giant (28 mm), symptomatic aneurysm, underwent FDD + C (Silk and 4 coils) procedure with immediate RROC class III. The last DSA was performed after SAH and revealed critical distal end stent stenosis and aneurysm remnant (RROC class III, OKM B).

### Six-month follow-up DSA

The complete aneurysm occlusion was demonstrated in 73.4% (Table 2). The results at the 6-month follow-up were similar in groups with and without coiling (RROC class I + II; 77.8% for FDD + C vs. 84.8% for FDD; *p*—not significant (NS)). At this stage 3 (5.6%) aneurysms with angiographic filling were qualified for retreatment. One stent thrombosis with moderate hemiparesis (mRS grade 3) occurred at the time of follow-up DSA procedure.

### Retreatment

Retreatment was needed for 3 (4.6%) residual aneurysms initially treated with FDD in 1 (2.2%), and FDD + C in 2 (10.5%, p-NS). In long-term follow-up all these aneurysms were totally occluded and the patients maintained a good neurological condition (OKM D, mRS 0).

Recanalisation of 1 (1.5%) aneurysm from OKM D to OKM B2 was demonstrated in DynaCT at the 3-month follow-up after treatment of 8 mm aneurysm with FDD + C (Silk and HydroSoft coils). Discontinuation of Clopidogrel administration was recommended and the 6-month follow-up DSA revealed the total aneurysm occlusion without additional treatment.

### Long-term radiological outcome

According to the OKM Scale, including the results of retreatment procedures, the most recent DSA showed no aneurysm filling (OKM D) in 62 (95.4%), entry remnant with no stasis (OKM C1) in 2 (3.1%) and subtotal filling with significant stasis (OKM B3) in 1 (1.5%) aneurysm which ruptured. Two aneurysms with entry remnant (OKM C1) were small, with a dome-to-neck ratio < 2, located close to the origin of the ophthalmic artery and were treated with FDD alone. The RROC class I or II was achieved in 98.5% of aneurysms, with insignificant difference between FDD + C and FDD groups (Table 2).

In total, the new FDD thrombosis or stenosis occurred in 5 (9.1%) out of 55 targeted ICAs in follow-up and was symptomatic in 3 (60%) of them. The last accessible follow-up angiograms demonstrated stent occlusion in 2 (3.8%), stenosis in 2 (3.8%) and normalisation of blood flow in 1 patient. The new failures occurred in 4 (16%) of the Silk FDDs and in 1 (4.5%) of the Pipeline FDDs. None of Fred and Surpass FDDs occluded or narrowed.

### Ophthalmic artery patency

The follow-up DSA demonstrated the ophthalmic artery occlusion in 6 (10.9%) out of 55 ICAs covered with FDDs. There was no visual deterioration in these patients.

### Complications

During 182 invasive procedures, including all DSAs and treatment sessions, 18 (34.6%) patients experienced 29 adverse events. The adverse events included: 3 complications following DSA, 5 failed treatment attempts, 19 procedural complications of completed treatment sessions and 2 access site complications (Table 3).

**Table 3:** Summary of complications.

	Number	Rate per invasive procedure (DSAs + treatment procedures) (N^o^ 182)	Rate per treatment procedure (N^o^ 63)	Rate per aneurysm (N^o^ 65)	Rate per patient (N^o^ 52)
Failed attempts of FDD deployment	5		7.9% (5/63)	7.7% (5/65)	9.6% (5/52)
**Procedural complications**	22*	10.4% (19/182)	25.4% (16/63)	30.8% (20/65)	30.8% (16/52)
Intraprocedural ICA dissection	3	1.6% (3/182)	3.2% (2/63)	4.6% (3/65)	5.8% (3/52)
Intraprocedural FDD thrombosis	4	2.2% (4/182)	4.8% (3/63)	4.6% (4/65)	7.7% (4/52)
Imperfect FDD expansion	8		12.7% (8/63)	12.3% (8/65)	15.4% (8/52)
Incorrect FDD distal end migration	3		4.8% (3/63)	4.6% (3/65)	5.7% (3/52)
Cerebral vasospasm	4	2.2% (4/182)	4.8% (3/63)	6.2% (4/65)	7.7% (4/52)
Aneurysms rupture	0				
Intracranial vessel perforation	0				
Intraparenchymal haemorrhage	0				
Access site complications	2	1.1% (2/182)	3.2% (2/63)	3.1% (2/65)	3.8% (2/52)
**Late complications**					
New FDD thrombosis/stenosis	5***	–	–	7.7%	9.6%
SAH from targeted aneurysm	1	–	–	1.5%	1.9%
Intraparenchymal haemorrhage	0	–	–	–	–
**Clinical sequelae (w/o visual complications)**					
Transient neurological deficit	2	1.1%	3.2%	3.1%	3.8%
Permanent morbidity	1	0.5%	1.6%	1.5%	1.9%
Mortality	1	–	–	1.5%	1.9%
Overall morbidity and mortality	2	–	–	3.1%	3.8%
**Visual complications**					
Cranial nerve III palsy	0	–	–	–	–
Permanent visual impairment	2	1.1%	3.2%	3.1%	3.8%
Visual loss	0	–	–	–	–

*Total number of adverse events of all DSAs and treatment procedures.

**55 ICAs were covered with FDDs, i.e. bilaterally in 3 cases.

***One late FDD thrombosis, that occurred during the follow-up DSA procedure, was also incorporated in “procedural complications”.

### DSA complications

Three adverse events of diagnostic or follow-up DSA included: asymptomatic FDD thrombosis, asymptomatic cerebral vasospasm and symptomatic ICA dissection.

### Procedural complications

Nineteen procedural complications in 14 (26.9%) patients occurred during treatment procedures and included: imperfect FDD expansion (8), incorrect migration of FDD distal end (3), FDD thrombosis (3), ICA dissection (2) and vasospasm (3). The procedural complications occurred in 18.2% (6/33) of patients treated with FDD alone and in 42.1% (8/19) treated with FDD + C (*p* = 0.1; the Fisher exact test).

The imperfect FDD expansion (Silk in 7 and Pipeline in 1) was treated with balloon angioplasty. The incorrect FDD (Silk in 1 and Pipeline in 2) migration demanded the device replacement in 1 and implantation of additional FDD in 1. The access site complications occurred in 2 cases and included femoral artery pseudoaneurysm (1) and thrombosis (1). Revision vascular surgery was needed in the latter case.

Additionally, in follow-up a new FDD stenosis developed spontaneously in 4 cases (Silk in 3 and Surpass in 1), which led to SAH in one. To sum up, the early or late complications occurred in 21 (40.4%) patients. Neurological sequelae of all complications were detailed in the chapter “Clinical outcomes” and are summarised in Table 3.

Success rates. Immediate technical success was defined as an accurate deployment of FDD, as it was planned, during the first procedure with no procedural complications. The immediate technical success rate was 70.7% (37/52) per patient and 70.8% (46/65) per aneurysm. The Immediate technical success rate was 76.1% (35/46) in FDD group and 57.9% (11/19) in FDD + C group (p-NS, the Fisher Exact Test).

Six months technical success was defined as an aneurysm occlusion greater than 90% (RROC class I–II) and normal blood flow through the ICA (no stenosis/dissection/occlusion) at the 6-month follow-up DSA. The 6-month technical success rate was 63.6% (35/52) per patient, 70.8% (46/65) per aneurysm, 71.7% (33/46) in the FDD group and 68.4% (13/19) in the FDD + C group (p-NS for FDD vs. FDD + C; the Fisher Exact Test).

Treatment success was defined as a conjunction of the following conditions: an aneurysm occlusion greater than 90% (RROC class I–II), no SAH from targeted aneurysm, mRS grade: 0–2 and no visual deterioration in the most recent follow-up. The treatment success rate was 94.2% (49/52) per patient, 95.4% (62/65) per aneurysm, 97.8% (45/46) in the FDD group and 89.5% (17/19) in the FDD + C group (p-NS for FDD vs. FDD + C, the Fisher Exact Test). However, the most desirable scenario (uneventful treatment procedure, no failed attempts, no retreatment procedures, RROC class I at 6 months DSA, uneventful clinical periprocedural and long-term course) materialised in 47.7% (30/65) of cases only. Potential predictors of the most favourable outcome are presented in Table 4.

**Table 4:** Uni- and multivariate analysis of potential predictors of the most desirable scenario (RROC class I at 6 months with uneventful treatment and follow-up course).

Variable	Total	Most desirable scenario /−/	Most desirable scenario/+/
	N^o^ (%) or mean ± SD	N^o^ (%) or mean ± SD	N^o^ (%) or mean ± SD
**Univariate analysis**			
Age*			
(y.o.)	52.3 ± 11.7	55.2 ± 11.0	49.0 ± 11.9
Sex			
F	57 (87.7%)	31 (88.6%)	26 (86.7%)
M	8 (12.3%)	4 (11.4%)	4 (13.3%)
Number of treated ICA-C6 UIAs			
Single	41 (63.1%)	21 (60%)	20 (66.7%)
Multiple	24 (36.9%)	14 (40%)	10 (33.3%)
Previous SAH from other aneurysm			
Not	61 (93.8%)	34 (97.1%)	27 (90.0%)
Yes	4 (6.2%)	1 (2.9%)	3 (10.0%)
Side			
Left	36 (55.4%)	19 (54.3%)	17 (56.7%)
Right	29 (44.6%)	16 (45.7%)	13 (43.3%)
Type/location			
ICA-blister like	2 (3.1%)	2 (5.7%)	0 (0.0%)
ICA C6a	39 (60%)	20 (57.1%)	19 (63.3%)
ICA C6b	24 (36.9%)	13 (37.1%)	11 (36.7%)
Aneurysm size			
< 7 mm	32 (49.2%)	17 (48.6%)	15 (50.0%)
> 7 mm	33 (50.8%)	18 (51.4%)	15 (50.0%)
Aneurysm size (mm)	8.8 ± 5.4	9.3 ± 6.0	8.3 ± 4.8
Neck size (mm)	4.7 ± 1.9	5.1 ± 1.9	4.3 ± 1.8
Dome-to-neck ratio	1.8 ± 0.7	1.8 ± 0.8	1.9 ± 0.7
Symptomatic UIA			
Not	57 (87.7%)	28 (80.0%)	29 (96.7%)
Yes	8 (12.3%)	7 (20.0%)	1 (3.3%)
Smoking			
Not	34 (52.3%)	16 (45.7%)	18 (60.0%)
Yes	31 (47.7%)	19 (54.3%)	12 (40.0%)
Diabetes mellitus			
Not	60 (92.3%)	31 (88.6%)	29 (96.7%)
Yes	5 (7.7%)	4 (11.4%)	1 (3.3%)
Arterial hypertension			
Not	33 (50.8%)	16 (45.7%)	17 (56.7%)
Yes	32 (49.2%)	19 (54.3%)	13 (43.3%)
Type of FDD*			
FRED	11 (16.9%)	2 (5.7%)	9 (30.0%)
PIPELINE	27 (41.5%)	12 (34.3%)	15 (50.0%)
SILK	26 (40%)	21 (60.0%)	5 (16.7%)
SURPASS	1 (1.5%)	0 (0.0%)	1 (3.3%)
Technique of treatment*			
FDD and coiling	19 (29.2%)	14 (40.0%)	5 (16.7%)
FDD alone	46 (70.8%)	21 (60.0%)	25 (83.3%)
Number of FDDs			
1	59 (90.8%)	31 (88.6%)	28 (93.3%)
> 1	6 (9.2%)	4 (11.4%)	2 (6.7%)

Variable	Crude OR (95% CI)	Adj. OR (95% CI)	
**Multivariate logistic regression (without treatment-related factors)**			
Age (continuous variable)	0.95 (0.91,1)	0.94 (0.89,0.99)	
Dome-to-neck ratio (continuous variable)	1.38 (0.71,2.69)	3.39 (1.23,9.39)	
Symptomatic aneurysm (Yes vs. Not)	0.14 (0.02,1.19)	0.01 (0,0.52)	

*Age (continuous variable), OR (95% CI): 0.95 (0.91,1); *p* = 0.038;

Technique of treatment, OR (95% CI): 3.33 (1.03,10.79), *p* = 0.045;

SILK FDD, OR (95% CI): 0.05 (0.01, 0.33), *p* = 0.002, and p-not significant for the rest of tested factors.

## Discussion

A shift toward the endovascular treatment of ICA-C6 aneurysms is noticeable^6,9–11^. Endovascular treatment shows some benefits over surgical clipping like a lower risk of visual and other neurologic complications^11,13,14^. However, more than one third of ICA-C6 UIAs are not completely occluded after traditional coiling with or without stenting^8^. That is why FDDs are now increasingly popular, but their effectiveness has to be proven in long-term evaluations. The risk of neurological deterioration immediately after treatment, beyond the visual symptoms, was 6% in our series. A satisfactory clinical outcome was achieved in 98.1% of patients. Permanent morbidity and mortality rates in similar series were: 0–4.5% and 0–1.4%, respectively^7,15,19,20^. The good general outcomes of our series are notably burdened by one catastrophic SAH from a treated aneurysm. Yet, our case of SAH reflects rather a problem of effectiveness of endovascular treatment in giant aneurysms. In this case the additional coiling was used, to promote the intra-aneurysmal thrombosis. However, only RROC class III was achieved. Probably, a denser coil packing should be attempted for such unstable aneurysms, for a more direct protection.

Visual improvement in symptomatic ICA-C6 aneurysms is also a goal of treatment. Indirect decompression with clinical improvement is possible after embolisation, because of a reduction in aneurysm pulsation^9,11,21^. Optimistic literature data indicates that even two thirds of patients may improve their vision after embolisation and three quarters after surgical clipping^4,12,13^. However, many reports do not include objective ophthalmological evaluation. On the contrary, more critical observations demonstrate that vision improvement is rare and at least one third of patients have posttreatment visual dysfunction^22,23^. Our experience on 8 symptomatic UIAs also showed a 25% risk of permanent vision deterioration and a little chance for improvement. Not only our experience indicates that visual symptoms may also appear in delayed period^24^. Nevertheless, the permanent visual impairment after treatment of asymptomatic UIA is unlikely to occur, based on our and other series^13^.

The immediate postprocedural complete obliteration rate was 4 times higher for aneurysms treated with FDD and coiling, but the mid- and long-term results were similar in the FDD and FDD + C groups. It indicates that FDDs alone are effective for most ICA-C6 UIAs and the additional coiling may be reasonable in selected cases.

The complete obliteration in 95.4% of aneurysms was finally achieved, as compared to 74.6–89.1% in other series^7,15,19,20,25^. The use of additional coiling in 29% of aneurysms could be the reason for better results in our series. Selection of aneurysms for additional coiling depended mainly upon the size and symptoms. Symptomatic aneurysms (5/8) and those larger than 12 mm (10/11) were qualified mainly for FDD + C.

The retreatment rate was 4.6% in our series and it ranges from 9 to 30% after coil embolisation and from 0.9 to 6.8% after flow-diversion in other series^4,8,19,20,25^. The revision “stent-in-stent” procedures were safe and fully effective in our patients.

Nowadays, we routinely use Dyna-CT imaging 3 months after FDD deployment. In our experience, the worst delayed complication is in-stent stenosis with persistent inflow to the aneurysm and the main purpose of doing Dyna-CT is to exclude this. The new FDD thrombosis or stenosis occurred in almost 10% of patients and all of them were diagnosed during the first half a year after treatment. Therefore, the close surveillance in this period is needed preferably with less invasive Dyna-CT, as one of adverse events was iatrogenic due to DSA procedure.

Occlusion of the ophthalmic artery was demonstrated in 11%, with no clinical sequalae. In the other series this proportion ranged from 3.6 to 21.6% with questionable clinical significance^3,19,23,26–28^.

About 40% of patients experienced complications that, not rarely, were multiple. The most frequent technical complication was imperfect FDD deployment (15% of patients), followed by failed attempts of FDD deployment and late FDD stenosis. Therefore, patients should be informed about the cumulative risk of all invasive procedures and the potentially delayed adverse events.

The procedural complications occurred in 42% of patients in the FDD + C group—more than twice as much as in the FDD group. Also, the immediate technical success rate was 18% lower in the FDD + C group than in the FDD group. Despite being insignificant, the differences should be pointed out for critical assessment whether the more complex treatment for more complex aneurysms is reasonable.

The treatment success was as high as 95% per aneurysm within a mean follow-up of 5 years. However, the most desirable scenario succeeded in less than half of aneurysms. This indicates that aggressive treatment brings very good final results but is burdened by considerable complication risks.

The multivariate analysis of demographic, clinical and morphometric factors demonstrated that younger age, higher dome-to-neck ratio and asymptomatic aneurysms favour the best outcomes.

Limitations of the study. Four different kinds of FDD had been used according to the needs and local availability. Furthermore, the UIAs treated with FDD alone significantly differed from those treated with FDD and coiling. Therefore our retrospective analysis cannot serve as a comparison of the different devices and techniques. Whether FDD with coiling is more effective than FDDs alone in complex aneurysms has to be proven in prospective trials.

## Conclusions

Flow-diverting devices, with the use of additional coiling in selected cases, may offer a very high proportion of satisfactory radiological and clinical outcomes. However, in our experience the high risk of complications of different type and time impose the need for critical assessment and for further improvement of technical capabilities of implanted devices.
